# The Effect of Cholesterol in SOPC Lipid Bilayers at Low Temperatures

**DOI:** 10.3390/membranes13030275

**Published:** 2023-02-26

**Authors:** Nikoleta Ivanova, Hassan Chamati

**Affiliations:** 1 Department of Physical Chemistry, University of Chemical Technology and Metallurgy, 8 Kliment Ohridski Blvd., 1756 Sofia, Bulgaria; 2 Institute of Solid State Physics, Bulgarian Academy of Sciences, 72 Tzarigradsko Chaussee Blvd., 1784 Sofia, Bulgaria

**Keywords:** membranes, SOPC lipid, cholesterol, atomistic molecular dynamics, Slipids Force Field

## Abstract

We study the behavior of lipid bilayers composed of SOPC (1-stearoyl-2-oleoyl-sn-glycero-3-phosphocholine) with different concentrations of cholesterol, ranging from 10 mol% to 50 mol% at 273 K. To this end, we carry out extensive atomistic molecular dynamic simulations with the aid of the Slipid force field aiming at computing basic bilayer parameters, as well as thermodynamic properties and structural characteristics. The obtained results are compared to available relevant experimental data and the outcome of atomistic simulations performed on bilayers composed of analogous phospholipids. Our results show a good quantitative, as well as qualitative, agreement with the main trends associated with the concentration increase in cholesterol. Moreover, it comes out that a change in the behavior of the bilayer is brought about at a concentration of about 30 mol% cholesterol. At this very concentration, some of the bilayer properties are found to exhibit a saturation and a significant long-range ordering of the lipid molecules in the membrane shows up.

## 1. Introduction

The basic structural units in cell membranes are lipid molecules [1,2]. They are diverse in conformation, with the main differences being in the type of the hydrophilic head, the length of the tails, and the saturation of the chains. Basically, biological membranes are composed of glycerophospholipids [3]. The most widespread type of lipids are PCs (phosphatidylcholines) that have hepatoprotective activity and participate in the reconstruction of the cell membrane surface [4]. Lipid membranes are known to exhibit structural phase transitions as a function of the temperature [1,5]. Depending on the alignment of the tails of the lipid molecules, the bilayer can be in three main phase states: solid-ordered $S_{O}$, liquid–crystal ($L_{\beta }$), or liquid disordered ($L_{D}$) [6,7]. The liquid–crystal state of membranes is called a *gel* and is not a standard solid state. The rate of lateral diffusion drops sharply when the bilayer transitions into the crystal state [8]. In the lipid bilayers, the presence of cholesterol leads to a liquid-ordered ($L_{O}$) phase state of the layers, which shares the characteristics of both gel and fluid phases [9]. The inclusion of cholesterol in a solid-ordered phase can lead to the disruption of the crystal lattice and reduce the order of the lipid chains [10]. One common (mono)unsaturated lipid from the PC group is 1-stearoyl-2-oleoyl-sn-glycero-3-phosphocholine (SOPC), whose molecule has a hydrophilic head and a glycerol ester bonding the two hydrophobic tails, known as saturated (sn-1) and unsaturated (sn-2) chains. The double bond in the sn-2 hydrocarbon chain leads to the formation of a kink. The properties of SOPC have been studied extensively by means of theoretical, as well as experimental, approaches. A schematic phase diagram of SOPC-cholesterol mixtures is proposed in Ref. [11]. It has been shown that, at low temperatures, the lipid molecules are in the solid-ordered $S_{O}$ state, and, by increasing the concentration of cholesterol, it undergoes a phase transition into the $L_{O}$ state. At temperatures slightly above 283 K, the lipid bilayer exhibits a $L_{D}$ state. For a recent review, the interested reader may consult Ref. [12].

Sterols are non-polar lipids possessing an inflexible structure due to the rings in their molecular conformation. The central representative in mammalian cells is cholesterol (cholest-5-en-3β-ol). Its presence in fluid membranes leads to stiffening of the bilayer, increased lipid ordering, and an increase in the thickness [12,13,14]. As a result of the methylation, the sterol tilt is reduced, thus promoting its ordering functions [9,13]. Moreover, the methylation determines the sterol’s orientations in the layer, depending on whether the lipid tails are saturated or unsaturated [15]. Another main reason for the stability and arrangement of lipid membranes is the formation of hydrogen bonds due to the inclusion of sucrose and cholesterol [16,17]. In membranes, cholesterol enters between the tails of the lipids and favors the trans-conformation of the chains [18]. It has been shown that the presence of cholesterol greatly affects the structural and mechanical properties of the SOPC lipid bilayer (see e.g., [12,19] and references therein).

A theoretical analysis of the behavior of lipid bilayers of different composition may be carried out with the aid of molecular dynamics (MD) [20,21,22,23]. Examining these biological systems at the atomic level gives a deeper insight on the mechanisms underlying their properties and functions. To this end, several force fields (FF) have been devised to model the interactions between the atoms of all building blocks. These are known to provide satisfactory results from the molecular simulation of membranes. One of the widely used force fields is CHARMM (Chemistry at HARvard Macromolecular Mechanics) [24], whose parametrization accounts for the balance of non-valent contacts describing the interactions between the lipid bilayer and the solvent. In CHARMM36 [25] field version, the correct area for saturated and unsaturated chains is reproduced without prior application of surface tension, enabling simulations of mixtures of different types of lipids, cholesterol, and proteins. Another FF used is Lipid14 (AMBER) [26]. This FF performs well in the NPT ensemble, but it is unable to reproduce the order parameter at the heads and the value for the second carbon atom of the sn-2 tail [27]. After two intermediate attempts [28,29] for developing force fields at zero surface tension in NPT, Stockholm lipids (Slipids) [30] for fully saturated acyl chains were devised. Unsaturated lipids can also be simulated via MD, exhibiting a good agreement with experimental structural measurements. The effect of cholesterol on these systems also agrees well with experimental findings. In the latest version of this FF [31], the dihedral angle calculation algorithm is optimized, and the results for the order parameter are improved. So far, most studies involving Slipid are carried out at relatively high temperatures [32,33,34]. In recent studies [19,35], we established that, even at low temperatures, the force field reproduces the structural characteristics of the cholesterol-free system and its counterpart with 10 mol% cholesterol fairly well.

In the present study, we carry out extensive atomistic MD simulations in conjunction with the Slipid force field to extract the thermodynamic properties and the structural behavior of the unsaturated SOPC, containing different concentrations of cholesterol, ranging from 10 to 50 mol%. To this end, we compute a number of physical quantities, such as the mass density distribution, the electrostatic potential, heat capacity, the order parameter and the tilt angle of the lipid tails, the area per lipid and the lateral diffusion coefficient, the radial distribution function, and the hydrogen bonding at low temperature in the vicinity of the experimental melting point of SOPC $T_{m}=279$ K [36]. We compare our results to available experimental measurements and data obtained with the aid of atomistic simulations performed on similar systems of other bilayers composed of analogous phospholipids. The other main objective of the present work is to check whether Slipid correctly predicts the basic properties of lipids at low temperatures, thus validating its transferability to other phases.

This paper is organized as follows: Section 2 gives details on the considered lipid systems and the used MD simulation procedure. Section 3 is devoted to the analysis of the outcome of the simulation and the behavior of the computed thermodynamic and structural quantities and discuss the obtained results along with a comparison to the available experimental and simulation data. This paper concludes with Section 4, where we summarize our results on molecular modeling of cholesterol-containing SOPC membranes.

## 2. Systems and Methods

The initial atomic coordinates in the systems composed of SOPC lipids and different cholesterol contents are generated with the aid of CHARMM-GUI [37,38,39,40]. The box dimensions in the *x* and *y* directions for each bilayer patch are different. They are scaled to fit the corresponding lipid areas, taking into account the amount of cholesterol [41,42,43]. On the other hand, it is known that cholesterol induces a significant reduction of the areas and shrinkage of the layer up to a certain critical concentration, above which the opposite effect sets in [39,44]. Then, the membrane swells, but its area does not reach the values corresponding to the unmixed systems [45]. This effect may be traced back to the smaller size of the cholesterol molecule.

**Figure 1:**
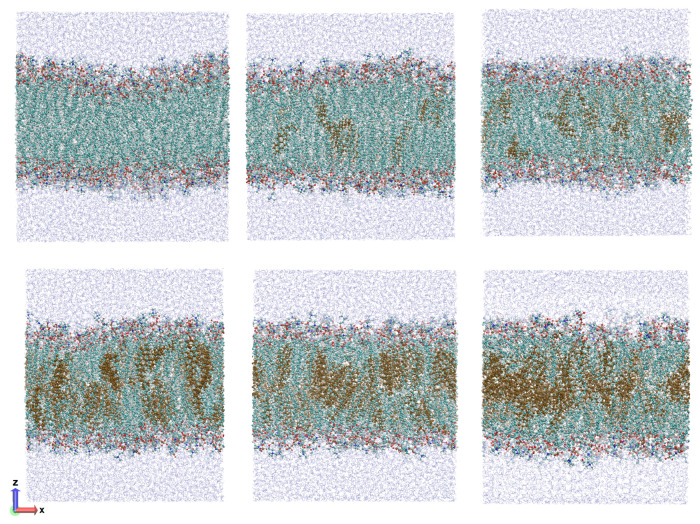
Side view of the initial configurations of the SOPC lipid bilayers at different cholesterol concentrations, shown in dark yellow. **Top left**—SOPC + 0% Chol, **top middle**—SOPC + 10% Chol, **top right**—SOPC + 20% Chol, **bottom left**—SOPC + 30% Chol, **bottom middle**—SOPC + 40% Chol and **bottom right**—SOPC + 50% Chol.

The constructed membranes contain 256 molecules—128 lipids in each monolayer. All systems are placed in cubic periodic boxes and subsequently hydrated adding enough water molecules, such that the size of all boxes is similar in the *z* direction. A detailed description of the composition of the studied systems and the size of the periodic box is presented in Table 1. Figure 1 shows the initial configurations for the system containing different concentration of cholesterol. Hereafter, the different systems will be designated as SOPC + n% Chol, where n (ranging from 0 to 50) corresponds to the amount, in mol%, of cholesterol in the membrane.

For the purpose of the present study, we do not make use of CHARMM-GUI to generate the coordinates of the water molecules added into the systems under considerations. We rather used GROMACS tools to achieve good hydration of the lipid heads and to avoid ending up with a sharp separation between the watery medium and the lipids. It is worth noting that some water molecules may be found in the vicinity of the first carbon atom of the glycerol ester, which is the more realistic scenario. Despite adding different number of water molecules, the thickness of the hydrated layer in all systems is large enough. Moreover, there is no interaction with the periodic image in the *z* direction, as the minimum width is about 5.4 nm for the pure system, i.e., SOPC + 0% Chol.

Atomistic molecular dynamics simulations were performed with the aid of Gromacs 2021.3 [46,47,48,49] in conjunction with Slipid 2020 force field [30,31], which is suitable for the description of lipids even at low temperatures [19]. The selected model for simulating water was TIP3P [50]. A leap-frog integrator [51] was used to integrate the equations of motion with a 2 fs timestep. For the description of the long-range electrostatic interactions, PME [52] with a cut-off of 1.2 nm is used. The same cut-off obtained with the Lennard-Jones potential was used for van der Waals interactions. The energy of the systems was minimized, then the systems were equilibrated up to 273 K in the NVT ensemble at about 10 ns. For a constant temperature, the V-rescale thermostat [53] was used in conjunction with the Berendsen barostat [54]. A posterior 400 ns long equilibrium run was carried out in the NPT ensemble at a temperature of 273 K and a pressure of 1 bar. A Nose-Hoover thermostat [55,56] was used to maintain a constant temperature. This thermostat is widely applied in membrane simulations and ensures a realistic movement of the atoms. An isotropic pressure scaling method was applied with a Parrinello-Rahman barostat [57,58]. Although the use of semi-isotropic pressure scaling is strongly recommended [59], it has been found to lead to unrealistic freezing [60]. Snapshots of the trajectory were saved at intervals of 10 ps. The statistical analyses were performed on trajectories over an additional 50 ns, corresponding to the production run of the simulations (400–450 ns period). In the analyzed trajectories, the coordinates are recorded each 2 ps. The visualization of the simulated membranes was achieved via VMD 1.9.4a [61]. For the statistical analyses of the trajectories, built-in tools in Gromacs were employed. MEMBPLUGIN tools in VMD [62], performing Voronoi analysis [63], were used to calculate the areas per lipids and the tilt angles of the hydrocarbon chains.

## 3. Results and Discussion

We computed a number of quantities to characterize the systems under consideration and to gain knowledge on the effect of cholesterol on the properties of SOPC. These include the bilayer density distribution, layer thickness, hydration of the heads, electrostatic potential, and the molar heat capacity of the membrane. Moreover, we assessed some structural parameters of the lipid molecules, such as the order parameter, tilt angles of the lipid tails relative to the bilayer normal, and dihedral angles for SOPC + 50% Chol system. Lipid mobility was determined by the lateral diffusion coefficient and the average area per lipid. The ability of the molecules to arrange in the bilayer was considered by calculating the radial distribution functions and the formation of hydrogen bonds, both in the membrane between lipids, as well as water molecules located on the surface of the bilayer. It is worth noting that there are insufficient experimental data and theoretical results in the literature on lipid bilayers at low temperatures. Thus, comparisons to experimental data are made only when possible and at temperatures different than those reported in this work. Moreover, results from similar molecular dynamics studies are discussed.

### 3.1. Bilayer Characteristics

#### 3.1.1. Mass Density Distribution

**Figure 2:**
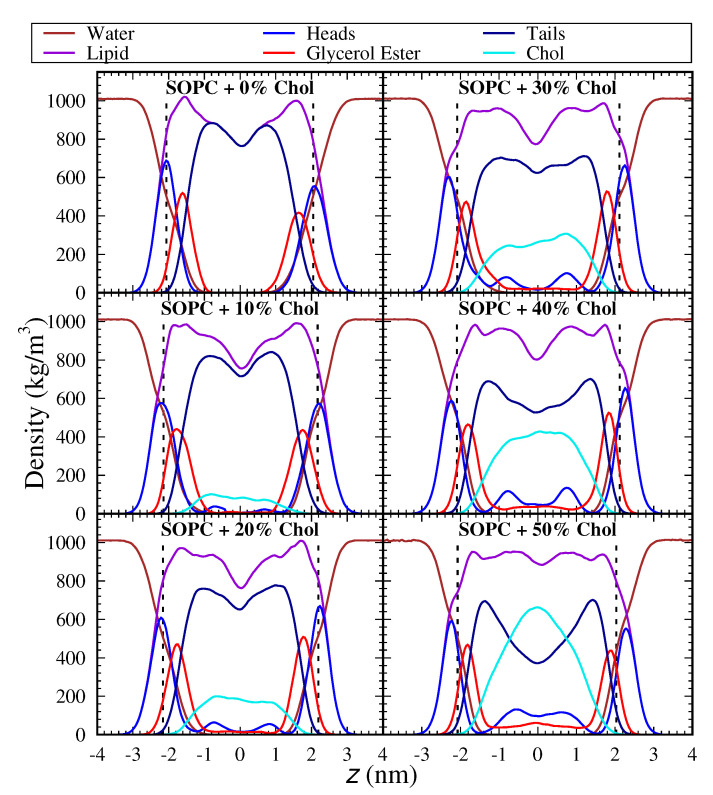
Mass density profiles in the *z* direction of the lipid bilayer, water, and the individual SOPC molecules. The dashed lines correspond to equimolar dividing surface (EDS) set at a water density of 500 kg·m${}^{-3}$.

The profiles and the mass density in the *z* direction (normal to the surface of the membrane) were calculated. The obtained data are presented in Figure 2 for all systems. From the obtained profiles, the thickness of the bilayer, submergence depth of the lipid heads into water and the thickness of the layer spanning the hydrophobic tails where water cannot be present, were determined. The corresponding data are presented in Table 2.

The profile corresponding to the distribution of the lipids in the membrane changes with increasing cholesterol concentration in the bilayer. In the system composed only of SOPC molecules, two peaks corresponding to the heads and the glycerol ester are clearly visible in Figure 2. A well defined minimum in the middle of the membrane is also present. When the amount of cholesterol increases, this minimum decreases and the peaks describing the position of the heads remain well pronounced. In the systems containing cholesterol, the location of the hydrophobic layer is also visible. At higher concentrations, its height is commensurate with the peak of the heads.

The profile shows that the distribution of the hydrophobic tails behaves differently with increasing cholesterol concentration compared to the total lipid profile. The SOPC + 30% Chol system differs significantly from the other systems. As the amount of cholesterol increases, the height of the profile decreases. At 30 mol%, the minimum in the middle of the membrane, corresponding to the ends of the tails, almost completely vanishes. At the two higher concentrations, the minimum reappears, being more pronounced for SOPC + 50% Chol. The two peaks in these profiles corresponding to the beginning of the tails are accounted for in the total lipid profile, as already mentioned.

The peaks showing the location of the heads and the glycerol ester in the lipid bilayer slightly diminish in heights with increasing cholesterol concentration. Their width increases, and the peaks are shifted towards the middle of the membrane. This effect may be traced back to the location of the cholesterol molecules in the membrane.

As expected, the profile’s change corresponds to a gradual increase in the amount of cholesterol in the systems. At the highest concentration, a pronounced peak is formed, which coincides with the minimum in the profile of the tails.

The equimolar dividing surface (EDS) is proposed by Gibbs [64], and this is a standard physico–chemical measure of the thickness of a bilayer. The EDS is determined at a density of 500 kg·m${}^{-3}$ of the aqueous phase for the two interfaces of the lipid bilayers. The bilayer thickness is defined as the difference in the positions of the two EDSs. The submergence depth of the lipid head in water is calculated from the thickness of the layer between the EDS and the rise of the peak of the heads. The hydrophobic layer of the membrane is enclosed by the profile of water at zero density [60,65]. Using block averaging, a standard error of 0.3 kg·m${}^{-3}$ for the lipid bilayers and 0.8 kg·m${}^{-3}$ for water were obtained.

The thickness of the lipid bilayer increases with the concentration of cholesterol to reach its highest value at 20 mol% Chol (see Table 2). Although the differences in values are close to the standard error, again the 30 mol% Chol system differs from the general trend. For this system, a slight shrinkage in the layer is noticeable, which can be seen in Figure 2. The thickness of the lipid bilayer remained almost the same at the next concentration, but, at 50 mol% Chol, the membrane shrinks again. The submergence depth of the hydrophilic heads in the aqueous phase shows the same increase with concentration as the bilayer thickness. Expectedly, for this quantity, too, the system with 30 mol% Chol differs and shows less hydration of the heads. The most substantial immersion occurs at the high concentration with the heads almost completely submerged in water. The thickness of the hydrophobic layer increases with cholesterol concentration. The increase is clearly associated with the amount of cholesterol, and the highest value was computed for 50 mol% Chol.

From the analysis above, it is clear that cholesterol strongly affects the thickness of the layer. Its location near the lipid heads can potentially affect their hydration. It is also found that, at 30 mol% Chol content, there is some saturation in the system, and the values of the considered quantities differ from the other systems.

The obtained thicknesses of the investigated membranes (4–5 nm) are within the expected range determined by experimental, as well as other molecular studies. Through molecular dynamics and the CHARM36 FF, thicknesses of 4.0 nm [66] and 4.1 nm [67] were found for cholesterol-free SOPC lipid bilayer. Experimental data at 283 K shows a thickness of about 4.08 nm [68]. Furthermore, the increase in lipid bilayer thickness with increasing cholesterol concentration has been confirmed by experimental data on other types of lipid bilayers [69,70]. Using X-ray lamellar diffraction at 303 K and 47 mol% cholesterol concentration, a bilayer thickness of 4.7 nm was found for SOPC [41]. An increase in the thickness of the lipid layer with the amount of cholesterol was also reported in Ref. [45]. The most significant increase is up to 30 mol%, after which the rates are maintained. The resulting thicknesses are close in linear size to those shown in Table 2. In a similar study, by varying cholesterol concentration again at 303 K at 50 mol% cholesterol content, a thickness of 4.8 nm was obtained [71].

#### 3.1.2. Electrostatic Potential

The profiles of the electrostatic potential were determined without taking into account periodic boundary conditions, and each frame of the trajectories was projected at the same center and in a box of the same size [72]. The electrostatic potential is calculated using Poisson’s equation [73].

**Figure 3:**
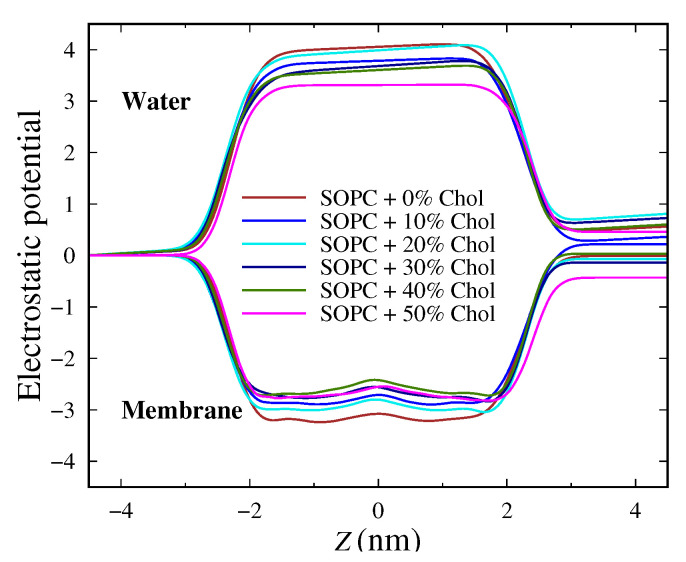
Electrostatic potential profiles in the direction normal to the bilayer surface. The top curves are relevant to the water molecules, while the bottom ones correspond to the lipid membrane.

The behavior of the electrostatic potential is shown in Figure 3. A slight decrease in the magnitude of the potential was reported with increasing cholesterol concentration. The hydroxyl group of cholesterol is directed towards the membrane surface. The molecule is located between the hydrophobic tails near the lipid heads. The profile corresponding to the lipids in the system containing 30 mol% and 50 mol% cholesterol completely overlapped, but there is a slight expansion of the potential. Furthermore, in the middle of the membranes, a minimum in the profile sets in. In general, the profile of the electrostatic potential is, to a large extent, identical to that of the mass density distribution for both lipids and the aqueous phase. It is worth mentioning that our electrostatic potential profile agrees fairly with the experimentally measured one [74].

#### 3.1.3. Isobaric Molar Heat Capacity

The heat capacity at constant pressure was calculated with the aid of the built in GROMACS software. For the purpose of this investigation, quantum corrections were not taken into account in the enthalpy. Moreover, the drift was subtracted. Our results (Table 3) show a gradual increase in the heat capacity with increasing amount of cholesterol up to 30 mol%. For higher concentrations, there is a steep increase in $C_{p}$. Again, we find that the system with 30 mol% cholesterol is peculiar, as the slope of $C_{p}$ changes at this concentration with $C_{p}=503$ J·mol${}^{-1}$·K${}^{-1}$.

It is well known that, at T=273 K, SOPC exhibits a gel (solid ordered) phase [36]. On the other hand, the addition of even a small amount of cholesterol increases the fludity of the lipid, thus driving lipid membranes to a liquid-ordered phase [9]. The behavior of the heat capacity obtained here confirms that the addition of cholesterol to an initially-ordered SOPC enforces the degree of lipid ordering, and no phase transition takes place.

For several membranes composed of different lipids (DPPC, DPPE, DPPA) at a temperature of 283 K, $C_{p}$ above 1200 J/mol·K was reported [75]. In the DMPC bilayer at 292 K, a heat capacity of the order of kJ was obtained [76]. The heat capacity as a function of temperature for a mixture of DOPC/DPPC (3:1) was examined and was around 273 K, and a value of $C_{p}$ of about 490 J/mol.K was reported [77]. Although the reported data are relevant to systems of different composition and at higher temperatures, the results obtained in the present study are consistent.

### 3.2. Structural Characteristics of the Lipids

#### 3.2.1. Order Parameter of the Lipid Tails

**Figure 4:**
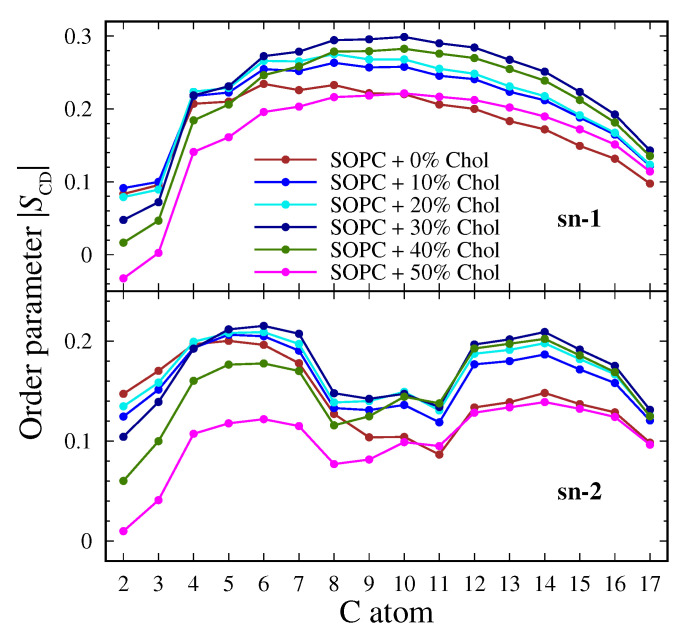
The standard order parameter $S_{CD}$ for SOPC lipid with the studied concentrations of cholesterol; sn-1 tail (**top**) and sn-2 tail (**bottom**).

The standard deuterium order parameter $S_{CD}$ was used to determine the order of the lipid tails and their inclination relative to the normal to the surface [78,79]. The results for the saturated (sn-1) and unsaturated (sn-2) tail at the studied cholesterol concentrations are shown in Figure 4.

Notice that the applied force field Slipid gives lower values than the experimentally determined ones [31]. Therefore, the analysis will concentrate on qualitative trends, rather than quantitative absolute values.

In the saturated tail, in the absence of cholesterol, the typical behavior of the order parameter is observed. A distinctly low value in the vicinity of the heads, presence of a kink between the second and third carbon atoms, and tail-end ordering are observed. The largest degree of arrangement at the end of the chain is reported for SOPC + 30% Chol. The most highly ordered system is SOPC + 30% Chol, and at the two largest concentrations, the system becomes disordered once more. As the cholesterol concentration increases, the kink near the head decreases, and the most disordered system is SOPC + 50% Chol, but the end of the tail still has a higher degree of order compared to the cholesterol-free system.

The order parameter for the unsaturated chain is similar for all systems, and the ends of the tails are equally ordered. The main distinctions are noticed close to the double bond, and the largest changes occur in the SOPC + 50% Chol system. At the sixth carbon atom, the formation of a kink is brought about with increasing cholesterol concentration. Again, the most ordered lipid bilayer contains 30 mol% cholesterol. At 40 mol% and 50 mol%, the tails are mostly disordered around the heads of the lipid. In general, the chain shows a larger degree of disorder than the saturated tail.

The presence of cholesterol in the systems affects the ordering of both tails that leads to an arrangement of lipid tails. The state of the membrane is expected to be liquid-ordered L${}_{O}$ or gel. The order parameter increases up to a concentration of 30 mol%. At the highest concentrations, the lipids are distorted, and the lowest values are reported at 50 mol% Chol. These results show the same tendency of saturation of some properties of the membranes, as reported above.

The Slipid force field used here gives a less-ordered system than the experimentally investigated one. A value of 0.33 at 303 K was reported for a cholesterol-free system with SOPC [45]. The increase in the degree of order with increasing cholesterol concentration was also confirmed for a DPPC layer [80]. Similar trends have been reported both in theoretical [81] and experimental studies [82]. For a three-component system of DSPC/DOPC/Chol [83], it has been reported that, at low concentrations of cholesterol, a solid state is realized. It is noteworthy that, in the present work, a critical concentration of cholesterol of 30 mol% was reported. A similar behavior of the order parameter has also been reported by an experimental study [45]. An increase in the parameter was observed at 50 mol% cholesterol, in contrast to our data. It should be noted that the named study considers the case from liquid disordered to liquid ordered state and not from solid to liquid ordered state, as is the case in the present work. It is clear from the phase diagram [11] that, in the presence of cholesterol in the system, the L${}_{O}$ phase is always reached. The subsequent increase in cholesterol leads to a decrease in the order in the lipid bilayers.

#### 3.2.2. Tilt Angles of the Lipid Tails

**Figure 5:**
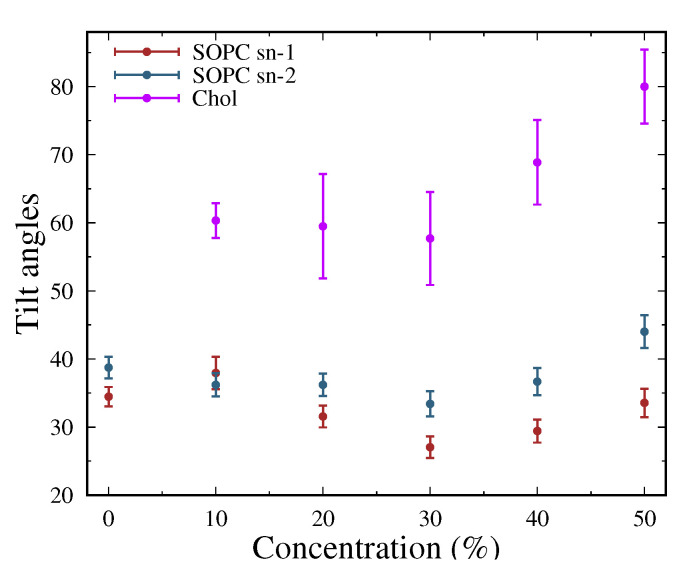
Tilt angles relative to the normal to the membrane surface for SOPC lipid tails (sn-1 and sn-2) and cholesterol for all systems.

Another important quantity that sheds light on the arrangement of the lipid tails is the tilt angle relative to the surface of the membrane. This characteristic was determined using the MEMBPLUGIN tool in VMD [62] by associating vectors to a lipid molecule. For the two tails, the first and last carbon atoms of the chain were taken as references, and for cholesterol, the oxygen atom of the hydroxyl group was chosen. The results with the corresponding standard errors are shown in Figure 5.

**Figure 6:**
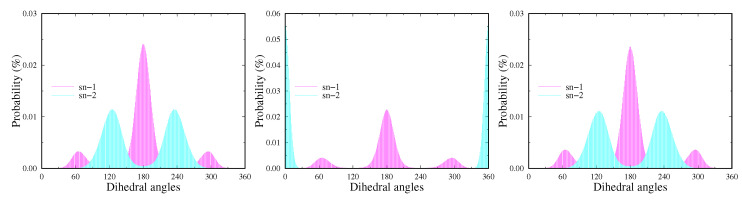
The most likely dihedral angles in the sn-2 tail around the double bond compared to the corresponding angle from the sn-1 tail computed for the SOPC + 50% Chol system.

The values obtained for the angles corresponding to the two tails are relatively close. Due to the presence of one double bond in the sn-2 tail, there is a variation in the angles for the two carbon chains, but their behavior with respect to cholesterol concentration is identical. As the cholesterol concentration increases, a difference in the angles shows up. The closest values for the two tails were found at 10 mol% Chol, and a larger difference was observed at 50 mol% Chol. There is a slight tendency for the angles to decrease with increasing concentration again up to 30 mol% Chol, after which the angles increase. Smaller angles correspond to more extended tails and closer to the normal. This means that some ordering occurs in the lipid bilayer in the systems. At the maximum concentration, the angles are the largest, i.e., the system exhibits a larger degree of disorder compared to the other concentrations. These results are in good agreement with the data obtained from the order parameter. Again, the values are close to those for the cholesterol-free system. The dihedral angles of the system SOPC + 50% Chol in the vicinity of the double bond are shown in Figure 6. In the applied version of the FF, their calculation method is optimized, bringing the values of $S_{CD}$ closer to the experimentally obtained ones. Expectedly, there is a large difference in the angles close to the double bond compared to the corresponding angles in the saturated tail.

The tilt angles of cholesterol is significantly different from that of lipid tails. The behavior reported for SOPC molecules occurs here, as well. The lowest values were observed for a system at 30 mol% Chol. Accordingly, in this system, cholesterol is maximally arranged.

Cholesterol affects the structural behavior of lipid molecules and leads to a highly ordered state of the membranes [9,12]. Similar to the previous group of analyses, the most significant differences from the general trend were reported for the system containing 30 mol% Chol. There, the arrangement of the hydrophobic tails is the strongest. At the higher concentrations, the angles were reported to increase to a degree close to that of the cholesterol-free system.

From the considerations made for the dependence between the length of the tail and the tilt angles, it is clear that the obtained values for SOPC are in line with experimental results [84]. Experimental tilt angle data were obtained for a wide range of lipids in different ratios [85]. The measured values for lipids are about 25${}^{\circ }$ and are close to the calculated values.

### 3.3. Area per Lipid and Lateral Diffusion Coefficient

**Figure 7:**
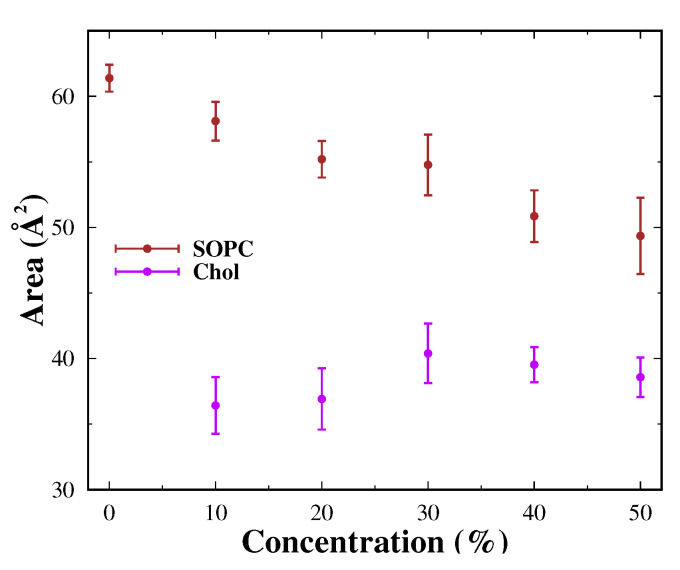
Average area per lipid of SOPC membranes at different cholesterol concentrations.

A fundamental feature of the phase behavior of the lipid bilayer is the mobility of the lipids in the membrane. Thus, to assess the degree of fluidity of the membrane, we computed the areas per lipid and the lateral diffusion coefficient. The obtained data are presented in Figure 7 and Table 4, along with the corresponding standard errors.

#### 3.3.1. Area per Lipid

To evaluate the average area of a lipid in a bilayer, we use the MEMBPLUGIN bundle in VMD [62]. It is based on the Voronoi analysis. Thus, a key atom is selected (say phosphorus for SOPC lipid and oxygen for the cholesterol). The coordinates of the selected atoms are projected onto a plane confined to a periodic box, and polygons are constructed on the plane with the aid of the qvoronoi program from the Qhull package [63]. The area of each polygon is calculated and averaged over the number of lipids of the respective species.

Average areas per lipid are affected by the presence of cholesterol in the membranes. It is noteworthy that the considered systems are at a low temperature (about 273 K), which implies smaller areas than those in the liquid disordered state [39,44]. In the cholesterol-free system, the average area per lipid of the SOPC is about 61.38 Å${}^{2}$. The lowest value for SOPC lipids was found at 50 mol% cholesterol content, and it is around 49.35 Å${}^{2}$. It is assumed that cholesterol leads to a reduction in areas. In the results shown, this tendency is noticeable. The areas of the initial structures (see Table 1) were predicted to decrease with increasing amounts of cholesterol. Therefore, the reported results are to some extent expected. The differences in the calculated areas are close to the standard deviation, but it can be strongly argued that the areas decrease with increasing cholesterol content. The resulting areas at higher cholesterol concentrations suggest a liquid-ordered state $L_{o}$ of the lipid bilayer. At these values, lipid mobility should be reduced.

In the case of cholesterol, the opposite trend is observed, and the areas per lipid increase. This is again due to the smaller size of the molecule, leading to the emergence of cavities in the bilayer. Average area values per lipid range from 36.50 Å${}^{2}$ to 40.00 Å${}^{2}$. The obtained values are again close to the standard error. It can be concluded that the change in the average area for cholesterol versus its concentration is relatively weak.

The obtained value for the area per lipid for the cholesterol-free system is in good agreement with that known in the literature [39,44]. At 303 K, a value of 62.7 Å${}^{2}$ was obtained for a number of monounsaturated lipids, depending on the length of the tail [86]. This area is higher than the calculated in the simulation, but the operating temperature is also high. With the aid of X-ray and neutron scattering at 293 K, an area of 63.8 Å${}^{2}$ [68] was determined for the SOPC monolayer. At the same temperature, values of about 61.4 Å${}^{2}$ were obtained for the bilayer [87,88]. The obtained value for the SOPC membrane in the present study is close to the experimentally found data. The reduction of areas with increasing cholesterol at physiological temperature is also confirmed for some monounsaturated lipids [89]. From short atomistic simulations carried out on a system of 64 molecules DPPC—cholesterol mixed bilayer, and the value of 45 Å${}^{2}$ for the area per molecule was obtained [90]. For a SOPC bilayer with 10 mol% cholesterol, the area determined by X-ray lamellar diffraction is 62 Å${}^{2}$ [41]. This value obtained at a different temperature is also slightly higher than our result.

#### 3.3.2. Lateral Diffusion Coefficient

The lateral diffusion coefficient, $D_{L}$, is calculated from Einstein’s relation [91] via the root-mean-square displacement of all atoms in the system. The movement of the lipids in the direction normal to the surface are excluded, and only the motion in the xy plane is taken into account. $D_{L}$ provides useful information on the mobility of lipid molecules in the lateral direction of the bilayer.

In all systems, the obtained values for the lateral diffusion coefficient are low, indicating a low mobility of the lipid heads in the membrane. The presence of cholesterol additionally hinders their movement. Moreover, taking into account the fact that the simulations are carried out at a low temperature (273 K), the systems appear to be somehow blocked, and practically no lateral movement of the lipids in the membrane takes place. This may be due to the tendency of the lipids to maintain their equilibrium position in the crystallized bilayer due to the resulting arrangement of the tails and tilting of the lipids relative to the *z*-axis. Furthermore, the liquid-ordered state $L_{O}$ of lipid bilayers is characterized by low values of lateral diffusion.

No clear trend in $D_{L}$ values could be observed with increasing cholesterol concentration. However, at large amounts, there is a certain increase in the diffusion coefficient, presumably due to the voids in the membrane caused by the small size of the cholesterol molecule. The values (Table 4) are within the standard deviation, and this increase has not been definitively confirmed.

The obtained values for the lateral diffusion coefficient are within the limits of the experimentally obtained ones. For the liquid ordered state of the lipids, the $D_{L}$ values are about 10${}^{-8}$ cm${}^{2}$.s${}^{-1}$ [6], and, in the gel state, they may reach up to 10${}^{-11}$ cm${}^{2}$.s${}^{-1}$ [8]. For other similar unsaturated lipids, DOPC and POPC, via NMR, at 298 K, D${}_{L}$ = 8·10${}^{-8}$ cm${}^{2}$.s${}^{-1}$ was obtained [92]. For mixed lipid bilayers with a united atom FF (MARTINI), an averaged over all lipids D${}_{L}$ about 10${}^{-9}$ cm${}^{2}$.s${}^{-1}$ at 295 K was obtained [93]. From NMR at 310 K for a mixture of PC lipids, PSM, and cholesterol, a lateral diffusion coefficient about 10${}^{-7}$ cm${}^{2}$.s${}^{-1}$ was measured [94]. This is another confirmation that the investigated SOPC membranes are in the $L_{O}$ state. The absence of a strong influence of cholesterol on the value of diffusion coefficient in mixed systems was noticed by measurements with the aid of NMR spectrometer [95].

### 3.4. Structural Behavior of the Membranes

#### 3.4.1. Radial Distribution Function

**Figure 8:**
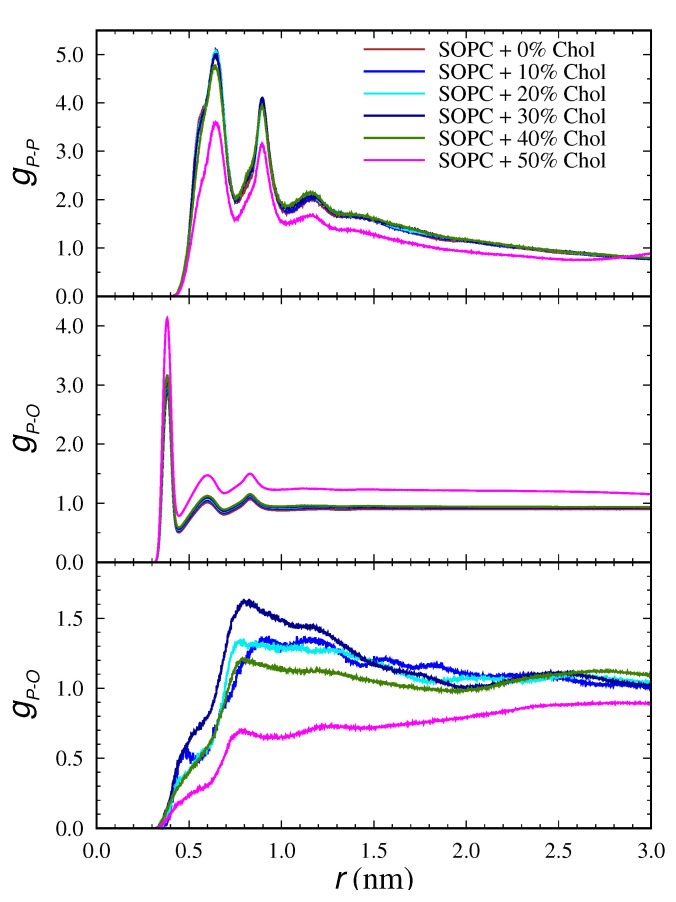
Radial distribution functions for the distances between *P-P* in SOPC lipids, *P-O* in SOPC and water, and *P-O* in SOPC and cholesterol at the different cholesterol concentrations.

The radial distribution function (RDF) describes the long-range order of any many-body system. To characterize the order in the studied lipid bilayers, RDF of the most likely distances between the phosphorus atoms in the heads between the lipids was calculated. Additionally, the cholesterol–lipid alignment was tracked by determining the distance between the phosphorus in the SOPC molecule and the oxygen atom in the cholesterol molecule. To determine the degree of ordering of the water molecules close to the surface, RDFs were calculated for the distance between the phosphorus atom of the hydrophilic head and the oxygen atom of water. The resulting profiles for the investigated systems are presented in Figure 8.

In the RDF profiles of *P-P* atoms in SOPC at all concentrations, three peaks with different heights are clearly defined. There are both short–(first peak)—and long range—(second and third peaks)—ordering of the heads. This finding is corroborated by the analysis of the parameters investigated above and more specifically by the lateral diffusion coefficient of the systems As the cholesterol concentration increases, the systems acquires higher degree of disorder, yet the liquid disordered phase $L_{d}$ remains beyond reach. The heights of the peaks in the 50 mol% Chol system is the lowest, with the third peak almost vanishing. The highest peak, i.e., ordering to a greatest extent, was observed with the cholesterol-free system and the one containing 30 mol%. As mentioned several times, at this critical concentration, saturation of some of the membrane properties occurs, and the calculated order parameter is highest (Figure 4).

Water molecules in the vicinity of a layer undergo some degree of arrangement, and a short-range order takes place. This has a weak consequence on the arrangement in the aqueous phase, as shown by the second and third peaks (Figure 8). With increasing cholesterol concentration, there is a slight increase in the height of the peaks, with the largest changes in the order of the molecules being reported in SOPC + 50% Chol. In this membrane, the hydrophilic heads were essentially in the water layer on the lipid layer (Figure 2).

No long-range order is observed in the arrangement of cholesterol with respect to SOPC lipid molecules (Figure 8). The main ordering is found between close molecules. Cholesterol is located between the hydrophobic tails, and a peak is reported in the profile. As the concentration of cholesterol increases, the arrangement of its molecules is preserved again, with the exception of the system SOPC + 30% Chol, where the height of the profile is the largest. In the case of the lipid bilayer with 50 mol% cholesterol, the profile is also different, and it shows the strongest disorder compared to the other systems.

#### 3.4.2. Hydrogen Bonds

Additional information about the order in the aqueous phase can be obtained from the amount of hydrogen bonds formed in the system. The average number of bonds between the corresponding oxygen atom from the hydrophilic head of SOPC and the hydroxyl group from the water molecule was calculated. The bonds among cholesterol and water are also considered. The data are presented in Table 5.

The distance for determining the hydrogen bonds between SOPC and water molecule is consistent with RDF profile, reaching a value up to 0.25 nm. The results show that, as the concentration of cholesterol increases, the distance for hydrogen bonds to form between SOPC and water molecules also increases. In the system, SOPC + 50% Chol, there is a strong interaction with the molecules near the surface of the membrane, which has an effect deep in the aqueous phase. As the distance increases, the average number of bonds decreases. However, the probability of their formation at larger distances increases.

For the interactions of cholesterol with water molecules, a distance of about 0.35 nm was chosen. The number of formed hydrogen bonds increases with increasing cholesterol concentration in the membranes. The slight increase may be traced back solely to the larger number of molecules that could take part in the interaction, and not to any change related to the ability to form hydrogen bonds. The distance and the probability of forming the bonds do not change significantly as the concentration increases. The probability of forming hydrogen bonds between cholesterol and water molecules is significantly lower compared to SOPC and water (not shown here). The main ordering of the water molecules near the membrane surface takes place due to the hydration of the lipid heads.

## 4. Summary

Cholesterol strongly affects the thickness of the lipid bilayer and the submergence depth of the hydrophilic heads. The obtained results agree well with the experimental data. In the SOPC + 30% Chol system, saturation of the layer is observed. The thickness of hydrophobic layer without the presence of water increases with the increase of cholesterol with no difference reported for the system with 30 mol% Chol. The resulting electrostatic potential profile is not significantly affected by the presence of cholesterol, but there is a slight decrease in its magnitude at higher concentrations. The profiles corresponding to the membrane at 30 mol% and 50 mol% cholesterol are almost completely overlapped. The heat capacity at constant pressure increases sharply (after SOPC + 30 % Chol) as the amount of cholesterol in the systems increases. The order parameter is affected by the concentration of cholesterol, with a maximum order of the lipid bilayer achieved at 30 mol% concentration. At a cholesterol concentration of 50 mol%, the membrane again becomes somewhat disordered, but it does not reach the values of the cholesterol-free system. The values of the tilt angles of the two tails are close, and, with the same dependence, the amount of cholesterol increases. The lowest reported value for the tilt angle of cholesterol is at 30 mol%, indicating that this is the most ordered system among those studied. Areas per lipid for SOPC molecule are greatly reduced with increasing cholesterol concentration in the membrane. The average areas per cholesterol molecule remain unchanged in the membranes. In the lateral diffusion coefficient, there is no clear trend due to the presence of cholesterol. From the calculated RDFs, the SOPC + 50 % Chol system differs most significantly, and from the height of the peaks, it is clear that the system with 30 mol% cholesterol shows the highest degree of ordering. To conclude, the behavior of all computed quantities indicates that all systems exhibit a liquid-ordered state. The system containing 30 mol% Chol appears to be critical, and saturation of most of the probed parameters was reported at higher concentrations.

## Figures and Tables

**Table 1:** Initial parameters of SOPC lipid bilayer with the studied concentrations of cholesterol. N is the number of molecules of the corresponding type. *A* is the initial area per lipid in *Å*${}^{2}$. *z* is the size in nm of the periodic box in the direction normal to the bilayer.

System	*N* – SOPC	*A* – SOPC	*N* – Chol	*A* – Chol	*N* – Water	*z*
SOPC + 0% Chol	256	66	0	0	13496	9.49
SOPC + 10% Chol	232	62	24	30	12,192	9.50
SOPC + 20% Chol	204	60	52	29	11,428	9.51
SOPC + 30% Chol	180	60	76	27	11,009	9.52
SOPC + 40% Chol	154	59	102	28	10,314	9.53
SOPC + 50% Chol	128	58	128	29	10,056	9.54

**Table 2:** Lipid bilayer parameters, in nm, determined from the calculated mass density profiles.

System	Bilayer Thickness	Submergence Depth	Hydrophobic Layer
SOPC + 0% Chol	4.11	1.19	0.59
SOPC + 10% Chol	4.31	1.25	0.78
SOPC + 20% Chol	4.35	1.41	0.98
SOPC + 30% Chol	4.19	1.32	1.13
SOPC + 40% Chol	4.21	1.37	1.56
SOPC + 50% Chol	4.10	1.43	1.81

**Table 3:** Molar heat capacity in J·mol${}^{-1}$·K${}^{-1}$ at constant pressure at different concentration of cholesterol.

System	$C_{p}$	Standard Error
SOPC + 0% Chol	134	25
SOPC + 10% Chol	180	64
SOPC + 20% Chol	278	53
SOPC + 30% Chol	504	38
SOPC + 40% Chol	1484	43
SOPC + 50% Chol	3492	20

**Table 4:** Lateral diffusion coefficient for SOPC, in $10^{-8}$ cm${}^{2}\cdot$s${}^{-1}$, of the SOPC membranes at different cholesterol concentrations.

System	Lateral Diffusion Coefficient	Standard Error
SOPC + 0% Chol	1.2	0.2
SOPC + 10% Chol	1.0	0.5
SOPC + 20% Chol	1.5	0.7
SOPC + 30% Chol	0.8	0.8
SOPC + 40% Chol	1.4	1.1
SOPC + 50% Chol	1.9	1.8

**Table 5:** Average number of hydrogen bonds between SOPC and water along with bonds between cholesterol and water for all considered systems.

System	SOPC–Water	Chol–Water
SOPC + 0% Chol	6.008 ± 2.448	–
SOPC + 10% Chol	5.612 ± 2.380	1.311 ± 1.187
SOPC + 20% Chol	5.346 ± 2.311	3.962 ± 2.014
SOPC + 30% Chol	4.834 ± 2.221	7.141 ± 2.621
SOPC + 40% Chol	4.534 ± 2.130	7.203 ± 2.719
SOPC + 50% Chol	3.991 ± 2.000	6.312 ± 2.526

## Data Availability

Not applicable.
